# Current Awareness on Comparative and Functional Genomics

**DOI:** 10.1002/cfg.119

**Published:** 2002-08


**Comparative and Functional Genomics**
*Comp Funct Genom* 2002; **3:** 389–396
Published online in Wiley InterScience (www.interscience.wiley.com). **DOI** 10.1002/cfg.119

### Current awareness on comparative and functional genomics

### 1 Reviews & symposia

2002. Special issue: Utilizing the genome sequence of parasitic protozoa. *Philos Trans R Soc Lond B* **357:** (1417)

Aardema MJ, MacGregor JT. 2002. Proctor & Gamble Co, Miami Valley Labs, POB 538707, Cincinnati, Oh 45253, USA. Toxicology and genetic toxicology in the new era of 'toxicogenomics': impact of '-omics' technologies. *Mutat Res* **499:** (1) 13.

Adams MD, Sekelsky JJ. 2002. Univ Nth Carolina, Dept Biol, 303 Fordham Hall, Chapel Hill, NC 27599, USA. From sequence to phenotype: Reverse genetics in *Drosophila melanogaster*. *Nat Rev Genet* **3:** (3) 189.

Allsop A, Illingworth R. 2002. AstraZeneca, Alderley Pk, Macclesfield SK10 4TG, England. The impact of genomics and related technologies on the search for new antibiotics. *J Appl Microbiol* **92:** (1) 7.

Altman RB, Klein TE. 2002. Stanford Med Informat, Stanford, Ca 94305, USA. Challenges for biomedical informatics and pharmacogenomics. *Annu Rev Pharmacol Toxicol* **42:** 113.

Andersen JS, Lyon CE, Fox AH, Leung AKL, Lam YW, Steen H, Mann M*, Lamond AI. 2002. *Univ Dundee, Wellcome Trust Bioctr, Dundee DD1 4HN, Scotland. Directed proteomic analysis of the human nucleolus (Review). *Curr Biol* **12:** (Suppl) 1.

Arcellana-Panlilio M, Robbins SM*. 2002. *Univ Calgary, Sthn Alberta Microarray Facil, 3330 Hosp Dr NW, Calgary, Alberta, Canada T2N 4N1. Cutting-edge technology: I. Global gene expression profiling using DNA microarrays. *Am J Physiol* **282:** (3) G397.

Arya P, Joseph R, Chou DTH. 2002. NRC Canada, Chem Biol Prog, 100 Sussex Rd, Ottawa, Ontario, Canada K1A 0R6. Toward high-throughout synthesis of complex natural product-like compounds in the genomics and proteomics age (Review). *Chem Biol* **9:** (2) 145.

Austin MJF, Kreiner T. 2002. Hoffmann La Roche Inc, 340 Kingsland St, Nutley NJ 07110, USA. Integrating genomics technologies in health care: Practice and policy challenges and opportunities. *Physiol Genomics* **8:** (1) 33.

Azuaje F. 2002. Univ Dublin - Trinity Coll, Dept Comp Sci, Dublin 2, Rep Ireland. Advancing post-genome data and system integration through machine learning (Conference Review). *Comp Funct Genom* **3:** (1) 28.

Barnes MR. 2002. GlaxoSmithKline Pharmaceut, Genet Bioinformatics, New Frontiers Sci Pk Nth, Third Ave, Harlow CM19 5AW, England. SNP and mutation data on the Web - Hidden treasures for uncovering (Review). *Comp Funct Genom* **3:** (1) 67.

Barnes S. 2002. Advanta, SES Europe NV, Ind Pk, Soldatenpl Z2 15, BE-3300 Tienen, Belgium. Comparing *Arabidopsis* to other flowering plants. *Curr Opin Plant Biol* **5:** (2) 128.

Beckers J, De Angelis MH. 2002. GSF, Natl Res Ctr Environm & Hlth, Inst Expt Genet, Ingolstaedter Landstr 1, DE-85764 Neuherberg, Germany. Large-scale mutational analysis for the annotation of the mouse genome. *Curr Opin Chem Biol* **6:** (1) 17.

Blackshaw S, Livesey R. 2002. Harvard Univ, Dept Genet, 200 Longwood Ave, Boston, Ma 02115, USA. Applying genomics technologies to neural development. *Curr Opin Neurobiol* **12:** (1) 110.

Brizuela L, Braun P, La Baer J*. 2001. *Harvard Univ, Inst Proteomics, 250 Longwood Ave, Boston, Ma 02115, USA. FLEXGene repository: From sequenced genomes to gene repositories for high-throughput functional biology and proteomics (Review). *Mol Biochem Parasitol* **118:** (2) 155.

Brussow H, Hendrix RW. 2002. Nestle Res Ctr, Vers-chez-les-Blanc, CH-1000 Lausanne 26, Switzerland. Phage genomics: Small is beautiful (Mini-Review). *Cell* **108:** (1) 13.

Buckler ES, Thornberry JM. 2002. USDA/ARS, Raleigh, NC 27695, USA. Plant molecular diversity and applications to genomics. *Curr Opin Plant Biol* **5:** (2) 107.

Camargo AA, De Souza SJ, Brentani RR, Simpson AJG. 2002. Ludwig Inst Canc Res, Rua Prof Antonio Prudente 109, BR-01509-010 Sao Paulo, Brazil. Human gene discovery through experimental definition of transcribed regions of the human genome. *Curr Opin Chem Biol* **6:** (1) 13.

Chung TP, Laramie JM, Province M, Cobb JP*. 2002. *Washington Univ, Dept Surg, 660 Euclid Ave, St Louis, Mo 63110, USA. Functional genomics of critical illness and injury. *Crit Care Med* **30:** (1 Suppl 1) S51.

Cirelli C. 2002. Univ Wisconsin, Dept Psychiat, 6001 Res Pk Blvd, Madison, Wi 53719, USA. How sleep deprivation affects gene expression in the brain: A review of recent findings. *J Appl Physiol* **92:** (1) 394.

Conrads TP, Issaq HJ, Veenstra TD*. 2002. *NCI, Analyt Chem Lab, SAIC Frederick, POB B, Bldg 469, Room 160, Frederick, Md 21702, USA. New tools for quantitative phosphoproteome analysis. *Biochem Biophys Res Commun* **290:** (3) 885.

Copley RR, Letunic I, Bork P. 2002. EMBL, Meyerhofstr 1, DE-69012 Heidelberg, Germany. Genome and protein evolution in eukaryotes. *Curr Opin Chem Biol* **6:** (1) 39.

Copley RR, Doerks T, Letunic I, Bork P*. 2002. *EMBL, Meyerhofstr 1, DE-69012 Heidelberg, Germany. Protein domain analysis in the era of complete genomes. *FEBS Lett* **513:** (1) 129.

Coppel RL. 2001. Monash Univ, Dept Microbiol, Melbourne, Vic 3800, Australia. Bioinformatics and the malaria genome: Facilitating access and exploitation of sequence information (Review). *Mol Biochem Parasitol* **118:** (2) 139.

Croston GE. 2002. Acadia Pharmaceut, 3911 Sorrento Valley Blvd, San Diego, Ca 92121, USA. Functional cell-based μHTS in chemical genomic drug discovery (Review). *Trends Biotechnol* **20:** (3) 110.

Davidson EH, Rast JP, Oliveri P, Ransick A, Calestani C, Yuh CH, Minokawa T, Amore G, Hinman V, Arenas-Mena C *et al*. 2002. Calif Inst Technol, Div Biol, Pasadena, Ca 91125, USA. A genomic regulatory network for development (Review). *Science* **295:** (5560) 1669.

Eiglmeier K, Simon S, Garnier T, Cole ST*. 2001. *Inst Pasteur, Unite Genet Mol Bacterienne, 28 rue Dr Roux, FR-75724 Paris 15, France. The integrated genome map of *Mycobacterium leprae*. *Lepr Rev* **72:** (4) 462.

Emmanuel E, Levy AA. 2002. Weizmann Inst Sci, Dept Plant Sci, IL-76100 Rehovot, Israel. Tomato mutants as tools for functional genomics. *Curr Opin Plant Biol* **5:** (2) 112.

Gilbert M, Albala JS. 2002. Lawrence Livermore Natl Lab, Biol & Biotechnol Res Prog, POB 5508, Livermore, Ca 94550, USA. Accelerating code to function: Sizing up the protein production line. *Curr Opin Chem Biol* **6:** (1) 102.

Glueck SB, Dzau VJ*. 2002. *Harvard Univ, Brigham & Women's Hosp, Dept Med, 75 Francis St, Boston, Ma 02115, USA. Physiological genomics: Implications in hypertension research. *Hypertension* **39:** (2 Pt 2 Suppl) 310.

Gmuender H. 2002. Genedata AG, Basel, Switzerland. Perspectives and challenges for DNA microarrays in drug discovery and development. *Biotechniques* **32:** (1) 152.

Goodman N. 2002. Address not available. Biological data becomes computer literate: New advances in bioinformatics. *Curr Opin Biotechnol* **13:** (1) 68.

In order to keep subscribers up-to-date with the latest developments in their field, this current awareness service is provided by John Wiley & Sons and contains newly-published material on comparative and functional genomics. Each bibliography is divided into 16 sections. 1 Reviews & symposia; 2 General; 3 Large-scale sequencing and mapping; 4 Evolutionary genomics; 5 Comparative genomics; 6 Pathways, gene families and regulons; 7 Pharmacogenomics; 8 EST, cDNA and other clone resources; 9 Functional genomics; 10 Transcriptomics; 11 Proteomics; 12 Protein structural genomics; 13 Metabolomics; 14 Genomic approaches to development; 15 Technological advances; 16 Bioinformatics. Within each section, articles are listed in alphabetical order with respect to author. If, in the preceding period, no publications are located relevant to any one of these headings, that section will be omitted.

Copyright © 2002 John Wiley & Sons, Ltd.

Granucci F, Castagnoli PR, Rogge L, Sinigaglia F*. 2001. *Roche Milano Ric, via Olgettina 58, IT-20132 Milan, Italy. Gene expression profiling in immune cells using microarray (Review). *Int Arch Allergy Immunol* **126:** (4) 257.

Greene AS. 2002. Med Coll Wisconsin, Dept Physiol, 8701 Watertown Plank Rd, Milwaukee, Wi 53226, USA. Application of physiological genomics to the microcirculation. *Microcirculation* **9:** (1) 3.

Grivet L, Arruda P*. 2002. *Univ Estadual Campinas, Ctr Biol Mol & Engn Genet, BR-13081-970 Campinas, SP, Brazil. Sugarcane genomics: Depicting the complex genome of an important tropical crop. *Curr Opin Plant Biol* **5:** (2) 122.

Grosset JH, Cole ST*. 2001. *Inst Pasteur, Unite Genet Mol Bacterienne, 28 rue Dr Roux, FR-75724 Paris 15, France. Genomics and the chemotherapy of leprosy. *Lepr Rev* **72:** (4) 429.

Gull K. 2002. Univ Manchester, Sch Biol Sci, 2-205 Stopford Bldg, Oxford Rd, Manchester M13 9PT, England. The cell biology of parasitism in *Trypansoma brucei*: Insights and drug targets from genomic approaches? *Curr Pharm Design* **8:** (4) 241.

Haberkorn U, Altmann A, Eisenhut M. 2002. Univ Heidelberg, Dept Nucl Med, Neuenheimer Feld 400, DE-69120 Heidelberg, Germany. Functional genomics and proteomics: The role of nuclear medicine. *Eur J Nucl Med* **29:** (1) 115.

Harwood CR, Moszer I. 2002. Univ Newcastle, Dept Microbiol & Immunol, Framlington Pl, Newcastle upon Tyne NE2 4HH, England. From gene regulation to gene function: Regulatory networks in *Bacillus subtilis* (Conference Review). *Comp Funct Genom* **3:** (1) 37.

Hawkins TL, Detter JC, Richardson PM. 2002. Joint Genome Inst, 2800 Mitchell Dr, Walnut Creek, Ca 94598, USA. Whole genome amplification: Applications and advances. *Curr Opin Biotechnol* **13:** (1) 65.

Hayaishi O. 2002. Osaka Biosci Inst, 6-2-4 Furuedai, Osaka 565 0874, Japan. Functional genomics of sleep and circadian rhythm: Molecular genetic studies on sleep-wake regulation, with special emphasis on the prostaglandin $D_2$ system (Review). *J Appl Physiol* **92:** (2) 863.

Hermjakob H, Apweiler R. 2002. Eur Bioinformatics Inst, Wellcome Trust Genome Campus, Hinxton, Cambridge CB10 1SD, England. TEMBLOR - Perspectives of EBI database services (Conference Review). *Comp Funct Genom* **3:** (1) 47.

Jacob HJ, Kwitek AE. 2002. Med Coll Wisconsin, Human & Mol Genet Ctr, 8701 Watertown Plank Rd, Milwaukee, Wi 53226, USA. Rat genetics: Attaching physiology and pharmacology to the genome. *Nat Rev Genet* **3:** (1) 33.

James P. 2001. Lund Univ, Wallenberg Lab 2, SE-22007 Lund, Sweden. Protein expression analysis: From 'tip of the iceberg' to a global method. *Dis Marker* **17:** (4) 235.

Jenkins S, Gibson N. 2002. AstraZeneca, R&D Genet, Mereside, Alderley Pk, Macclesfield SK10 4TG, England. High-throughput SNP genotyping (Review). *Comp Funct Genom* **3:** (1) 57.

Karst U, REALIS Consortium. 2002. GBF, Mascheroder Weg 1, DE-38124 Braunschweig, Germany. REALIS: Postgenomic analysis of *Listeria monocytogenes* (Conference Review). *Comp Funct Genom* **3:** (1) 32.

Kennelly PJ. 2002. Virginia Polytech Inst & State Univ, Dept Biochem, Blacksburg, Va 24061, USA. Protein kinases and protein phosphatases in prokaryotes: A genomic perspective (Minireview). *FEMS Microbiol Lett* **206:** (1) 1.

Kloos DU, Choi C, Wingender E. 2002. BIOBASE GmbH, Halchtersche Str 33, DE-38304 Wolfenbuttel, Germany. The TGF-β-Smad network: Introducing bioinformatic tools (Review). *Trends Genet* **18:** (2) 96.

Korke R, Rink A, Seow TK, Chung MCM, Beattie CW, Hu WS*. 2002. *Univ Minnesota, Dept Chem Engn & Mat Sci, 421 Washington Ave SE, Minneapolis, Mn 55455, USA. Genomic and proteomic perspectives in cell culture engineering. *J Biotechnol* **94:** (1) 73.

Lechner D, Lathrop GM, Gut IG. 2002. Ctr Natl Genotypage, 2 rue Gaston Cremieux, FR-91057 Evry, France. Large-scale genotyping by mass spectrometry: Experience, advances and obstacles. *Curr Opin Chem Biol* **6:** (1) 31.

Li XM, Gu WK, Mohan S, Baylink DJ*. 2002. *Pettis Mem VAMC, Div Mol Genet, 11201 Benton St, Loma Linda, Ca 92357, USA. DNA microarrays: Their use and misuse. *Microcirculation* **9:** (1) 13.

Lilley KS, Razzaq A, Dupree P. 2002. Univ Cambridge, Dept Biochem, Downing Site, Cambridge CB2 1QW, England. Two-dimensional gel electrophoresis: Recent advances in sample preparation, detection and quantitation. *Curr Opin Chem Biol* **6:** (1) 46.

Ma DH, Li M*. 2001. *Johns Hopkins Univ, Dept Physiol, 725 Nth Wolfe St, Baltimore, Md 21205, USA. Applications of display technologies to proteomic analyses. *J Cell Biochem* **84:** (Suppl 37) 34.

MacCoss MJ, Yates JR. 2001. Scripps Clin & Res Inst, Dept Cell Biol, 10550 Nth Torrey Pines Rd, La Jolla, Ca 92122, USA. Proteomics: Analytical tools and techniques. *Curr Opin Clin Nutr Metab Care* **4:** (5) 369.

Meyerowitz EM. 2002. Calif Inst Technol, Div Biol, Pasadena, Ca 91125, USA. Comparative genomics: Plants compared to animals: The broadest comparative study of development (Review). *Science* **295:** (5559) 1482.

Mirzabekov A, Kolchinsky A. 2002. Russian Acad Sci, Engelhardt Inst Mol Biol, Vavilova St 32, RU-119991 Moscow, Russia. Emerging array-based technologies in proteomics. *Curr Opin Chem Biol* **6:** (1) 70.

Mouradian S. 2002. Caliper Technol Corp, 605 Fairchild Dr, Mount View, Ca 94043, USA. Lab-on-a-chip: Applications in proteomics. *Curr Opin Chem Biol* **6:** (1) 51.

Mousses S, Kallioniemi A, Kauraniemi P, Elkahloun A, Kallioniemi OP. 2002. NIH/NHGRI, Canc Genet Branch, Bethesda, Md 20892, USA. Clinical and functional target validation using tissue and cell microarrays. *Curr Opin Chem Biol* **6:** (1) 97.

Mullet JE, Klein RR, Klein PE. 2002. Texas A&M Univ, Inst Plant Genomics & Biotechnol, College Station, Tx 77843, USA. *Sorghum bicolor*: An important species for comparative grass genomics and a source of beneficial genes for agriculture. *Curr Opin Plant Biol* **5:** (2) 118.

Newmark PA, Alvarado AS. 2002. Univ Illinois, Dept Cell & Struct Biol, 601 Sth Goodwin Ave, Urbana, Il 61801, USA. Not your father's planarian: A classic model enters the era of functional genomics. *Nat Rev Genet* **3:** (3) 210.

Nolan JP, Mandy FF. 2001. Los Alamos Natl Lab, Natl Flow Cytometry Resources, Los Alamos, NM 87545, USA. Suspension array technology: New tools for gene and protein analysis. *Cell Mol Biol (Noisy-le-Grand)* **47:** (7) 1241.

Norin M, Sundstrom M. 2002. Biovitrum, Dept Struct Chem, Stockholm, Sweden. Structural proteomics: Developments in structure-to-function predictions (Review). *Trends Biotechnol* **20:** (2) 79.

Oliver DJ, Nikolau B, Wurtele ES. 2002. Iowa State Univ Sci & Technol, Dept Bot, Ames, Ia 50011, USA. Functional genomics: High-throughput mRNA, protein, and metabolite analyses. *Metab Eng* **4:** (1) 98.

Osterlund MT, Paterson AH. 2002. Univ Georgia, Ctr Appl Genet Technol, Athens, Ga 30602, USA. Applied plant genomics: The secret is integration. *Curr Opin Plant Biol* **5:** (2) 141.

Panisko EA, Conrads TP, Goshe MB, Veenstra TD*. 2002. *NCI, SAIC Frederick, POB B, Bldg 469, Rm 160, Frederick, Md 21702, USA. The postgenomic age: Characterization of proteomes. *Exp Hematol* **30:** (2) 97.

Patton WF, Beechem JM. 2002. Mol Probes Inc, Proteomics Sect, 4849 Pitchford Ave, Eugene, Or 97402, USA. Rainbow's end: The quest for multiplexed fluorescence quantitative analysis in proteomics. *Curr Opin Chem Biol* **6:** (1) 63.

Pellegrini M, Thompson M, Fierro J, Bowers P. 2001. Protein Pathways, 1145 Gayley Ave, Los Angeles, Ca, USA. Computational method to assign microbial genes to pathways. *J Cell Biochem* **84:** (Suppl 37) 106.

Phelps TJ, Palumbo AV, Beliaev AS. 2002. Oak Ridge Natl Lab, Div Environm Sci, Oak Ridge, Tn 37831, USA. Metabolomics and microarrays for improved understanding of phenotypic characteristics controlled by both genomics and environmental constraints. *Curr Opin Biotechnol* **13:** (1) 20.

Rappsilber J, Mann M. 2002. Univ Sthn Denmark, Lab Ctr Expt Bioinformat, Campusvej 55, DK-5230 Odense M, Denmark. What does it mean to identify a protein in proteomics? *Trends Biochem Sci* **27:** (2) 74.

Rathod PK, Ganesan K, Hayward RE, Bozdech Z, De Risi JL. 2002. Univ Washington, Dept Chem, Seattle, Wa 98195, USA. DNA microarrays for malaria. *Trends Parasitol* **18:** (1) 39.

Reidhaar-Olson JF, Rhees BK, Hammer J. 2001. Hoffmann La Roche Inc, Dept Gen & Informat Sci, 340 Kingsland St, Nutley, NJ 07110, USA. Genomics approaches to drug discovery. *J Cell Biochem* **84:** (Suppl 37) 110.

Rice P, Jassal B, De Daruvar A. 2002. LION Biosci Ltd, Compass Hse, 80-82 Newmarket Rd, Cambridge CB5 8DZ, England. RIBDB: An SRS based infrastructure for REALIS (Conference Review). *Comp*

Copyright © 2002 John Wiley & Sons, Ltd. 

*Funct Genom* **3:** (1) 35.
Ronaghi M, Elahi E. 2002. Stanford Univ, Genome Technol Ctr, 855 California Ave, Palo Alto, Ca 94304, USA. Discovery of single nucleotide polymorphisms and mutations by pyrosequencing (Review). *Comp Funct Genom* **3:** (1) 51.
Sassetti C, Rubin EJ. 2002. Harvard Univ, Dept Immunol & Infect Dis, 667 Huntington Ave, Boston, Ma 02115, USA. Genomic analyses of microbial virulence. *Curr Opin Microbiol* **5:** (1) 27.
Schoolnik GK. 2002. Stanford Univ, Beckman Ctr, Stanford, Ca 94305, USA. Functional and comparative genomics of pathogenic bacteria. *Curr Opin Microbiol* **5:** (1) 20.
Schweitzer B, Kingsmore SF. 2002. Mol Staging Inc, 300 George St, New Haven, Ct 06511, USA. Measuring proteins on microarrays. *Curr Opin Biotechnol* **13:** (1) 14.
Shields DC, O'Halloran AM. 2002. Roy Coll Surg Ireland, Dept Clin Pharmacol, 123 St Stephen's Green, Dublin 2, Rep Ireland. Integrating genotypic data with transcriptomic and proteomic data (Conference Review). *Comp Funct Genom* **3:** (1) 22.
Stephenson JL, McLuckey SA, Reid GE, Wells JM, Bundy JL. 2002. Res Triangle Inst, 3040 Cornwallis Rd, Res Triangle Park, NC 27709, USA. Ion/ion chemistry as a top-down approach for protein analysis. *Curr Opin Biotechnol* **13:** (1) 57.
Stevens R. 2002. Univ Manchester, Dept Comp Sci, Oxford Rd, Manchester M13 9PL, England. Ontology based document enrichment in bioinformatics (Conference Review). *Comp Funct Genom* **3:** (1) 42.
Stoll D, Templin MF, Schrenk M, Traub PC, Vohringer CF, Joos TO*. 2002. *Univ Tubingen, NMI Nat & Med Sci Inst, Markwiesenstr 55, DE-72770 Reutlingen, Germany. Protein microarray technology. *Front Biosci* **7:** (Jan) C13.
Strosberg AD. 2001. Hybrigen SA, 3-5 Impasse Reille, FR-75014 Paris, France. Functional proteomics to exploit genome sequences. *Cell Mol Biol (Noisy-le-Grand)* **47:** (8) 1295.
Thalmann I. 2001. Washington Univ, Dept Otolaryngol, 660 Sth Euclid Ave, St Louis, Mo 63110, USA. Proteomics and the inner ear. *Dis Marker* **17:** (4) 259.
Thompson J, Janse CJ, Waters AP*. 2001. *Leiden Univ, Dept Parasitol, NL-2300 RC Leiden, The Netherlands. Comparative genomics in *Plasmodium*: A tool for the identification of genes and functional analysis (Review). *Mol Biochem Parasitol* **118:** (2) 147.
Uetz P. 2002. Forschungszentrum, Inst Toxikol & Genet, Postfach 3640, DE-76021 Karlsruhe, Germany. Two-hybrid arrays. *Curr Opin Chem Biol* **6:** (1) 57.
Warner EE, Dieckgraefe BK*. 2002. *Washington Univ, Div Gastroenterol, 660 Sth Euclid Ave, St Louis, Mo 63110, USA. Application of genome-wide gene expression profiling by high-density DNA arrays to the treatment and study of inflammatory bowel disease (Review). *Inflamm Bowel Dis* **8:** (2) 140.
Weinberger SR, Dalmasso EA, Fung ET. 2002. Ciphergen Biosyst, 6611 Dumbarton Circle, Fremont, Ca 94555, USA. Current achievements using ProteinChip® array technology. *Curr Opin Chem Biol* **6:** (1) 86.
Wheeler PR. 2001. Vet Labs Agcy, TB Res Unit, Weybridge KT15 3NB, England. The microbial physiologist's guide to the leprosy genome. *Lepr Rev* **72:** (4) 399.
Wild CP, Law GR, Roman E. 2002. Univ Leeds, Mol Epidemiol Unit, Firth Bldg, Leeds LS2 9JT, England. Molecular epidemiology and cancer: Promising areas for future research in the post-genomic era. *Mutat Res* **499:** (1) 3.
Wilson DS, Nock S. 2002. Zyomyx Inc, 26101 Res Rd, Hayward, Ca 94545, USA. Functional protein microarrays. *Curr Opin Chem Biol* **6:** (1) 81.
Zheng XFS, Chan TF. 2002. Univ Washington, Cell & Mol Biol Prog, Campus Box 8069, St Louis, Mo 63110, USA. Chemical genomics in the global study of protein functions (Review). *Drug Discov Today* **7:** (3) 197.

### 3 Large-scale sequencing and mapping

Coe E, Cone K, McMullen M, Chen SS, Davis G, Gardiner J, Liscum E, Polacco M, Paterson A, Sanchez-Villeda H, Soderlund C, Wing R. 2002. Univ Missouri, Dept Agron, Columbia, Mo 65211, USA. Access to the maize genome: An integrated physical and genetic map. *Plant Physiol* **128:** (1) 9.
Del Vecchio VG, Kapatral V, Redkar RJ, Patra G, Mujer C, Los T, Ivanova N, Anderson I, Bhattacharyya A, Lykidis A *et al*. 2002. Univ Scranton, Inst Mol Biol & Med, Scranton, Pa 18510, USA. The genome sequence of the faculative intracellular pathogen *Brucella melitensis*. *Proc Natl Acad Sci U S A* **99:** (1) 443.
Schulte U, Becker I, Mewes HW, Mannhaupt G. 2002. Univ Dusseldorf, Inst Biochem, DE-40225 Dusseldorf, Germany. Large scale analysis of sequences from *Neurospora crassa*. *J Biotechnol* **94:** (1) 3.
Wood V, Gwilliam R, Rajandream MA*, Lyne M, Lyne R, Stewart A, Sgouros J, Peat N, Hayles J *et al*. 2002. *Wellcome Trust Sanger Inst, Wellcome Trust Genome Campus, Cambridge CB10 1SA, England. The genome sequence of *Schizosaccharomyces pombe*. *Nature* **415:** (6874) 871.
Zabarovska VI, Gizatullin RZ, Al Amin AN, Podowski R, Protopopov AI, Lofdahl S, Wahlestedt C, Winberg G, Kashuba VI, Ernberg I, Zabarovsky ER*. 2002. *Karolinska Inst, Ctr Microbiol & Tumor Biol, SE-17177 Stockholm, Sweden. A new approach to genome mapping and sequencing: Slalom libraries (Electronic paper). *Nucleic Acids Res* **30:** (2) E6.

### 4 Evolutionary genomics

Dai LX, Zimmerly S*. 2002. *Univ Calgary, Dept Biol Sci, 2500 Univ Dr 1 NW, Calgary, Alberta, Canada T2N 1N4. Compilation and analysis of group II intron insertions in bacterial genomes: Evidence for retroelement behavior. *Nucleic Acids Res* **30:** (5) 1091.
Fay JC, Wyckoff GJ, Wu CI. 2002. Univ Calif, Lawrence Berkeley Lab, Dept Genome Sci, Berkeley, Ca 94720, USA. Testing the neutral theory of molecular evolution with genomic data from *Drosophila*. *Nature* **415:** (6875) 1024.
Hansen-Wester I, Stecher B, Hensel M*. 2002. *Univ Erlangen Nurnberg, Inst Klin Mikrobiol Immunol & Hyg, Wasserturmstr 3-5, DE-91054 Erlangen, Germany. Analyses of the evolutionary distribution of *Salmonella* translocated effectors. *Infect Immun* **70:** (3) 1619.
Hartman H, Fedorov A. 2002. MIT, Dept Biol, 77 Massachusetts Ave, Cambridge, Ma 02139, USA. The origin of the eukaryotic cell: A genomic investigation. *Proc Natl Acad Sci U S A* **99:** (3) 1420.
Krakauer DC, Plotkin JB. 2002. Santa Fe Inst, 1399 Hyde Pk Rd, Santa Fe, NM 87501, USA. Redundancy, antiredundancy, and the robustness of genomes. *Proc Natl Acad Sci U S A* **99:** (3) 1405.

### 5 Comparative genomics

Bapteste E, Brinkmann H, Lee JA, Moore DV, Sensen CW, Gordon P, Durufle L, Gaasterland T, Lopez P, Muller M, Philippe H*. 2002. *Univ Paris 6, CNRS/UMR 7622, 9 Quai St Bernard, FR-75005 Paris, France. The analysis of 100 genes supports the grouping of three highly divergent amoebae: *Dictyostelium, Entamoeba*, and *Mastigamoeba*. *Proc Natl Acad Sci U S A* **99:** (3) 1414.
Carlton JMR, Muller R, Yowell CA, Fluegge MR, Sturrock KA, Pritt JR, Vargas-Serrato E, Galinski MR, Barnwell JW, Mulder N, Kanapin A, Cawley SE, Hide WA, Dame JB. 2001. Inst Genomic Res, Med Ctr Dr, Rockville, Md 20850, USA. Profiling the malaria genome: A gene survey of three species of malaria parasite with comparison to other apicomplexan species. *Mol Biochem Parasitol* **118:** (2) 201.
Dziejman M, Balon E, Boyd D, Fraser CM, Heidelberg JF, Mekalanos JJ*. 2002. *Harvard Univ, Dept Microbiol & Mol Genet, Boston, Ma 02115, USA. Comparative genomic analysis of *Vibrio cholerae*: Genes that correlate with cholera endemic and pandemic disease. *Proc Natl Acad Sci U S A* **99:** (3) 1556.
Gu ZL, Cavalcanti A, Chen FC, Bouman P, Li WH*. 2002. *Univ Chicago, Dept Ecol & Evolut, 1101 East 57th St, Chicago, Il 60637, USA. Extent of gene duplication in the genomes of *Drosophila*, nematode, and yeast. *Mol Biol Evol* **19:** (3) 256.
Sebban M, Mokrousov I, Rastogi N, Sola C*. 2002. *Inst Pasteur Guadeloupe, Unite TB & Mycobacteries, FR-97165 Pointe-a-Pitre, Guadeloupe. A data-mining approach to spacer oligonucleotide typing of *Mycobacterium tuberculosis*. *Bioinformatics* **18:** (2) 235.
Thomas JW, Touchman JW*. 2002. *NIH/NHGRI, Genome Technol Branch, Bethesda, Md 20892, USA. Vertebrate genome sequencing: Building a backbone for comparative genomics. *Trends Genet* **18:** (2) 104.

Copyright © 2002 John Wiley & Sons, Ltd. 

### 6 Pathways, gene families and regulons

Shmulevich I, Dougherty ER, Kim S, Zhang W. 2002. Univ Texas, Canc Genomics Lab, 1515 Holcombe Blvd, Houston, Tx 77030, USA. Probabilistic Boolean networks: A rule-based uncertainty model for gene regulatory networks. *Bioinformatics* **18:** (2) 261.

Wagner A. 2002. Univ New Mexico, Dept Biol, Santa Fe Inst, Albuquerque, NM 87131, USA. Estimating coarse gene network structure from large-scale gene perturbation data. *Genome Res* **12:** (2) 309.

### 7 Pharmacogenomics

Belbin TJ, Singh B, Barber I, Socci N, Wenig B, Smith R, Prystowsky MB, Childs G*. 2002. *Montefiore Med Ctr, Dept Mol Genet, 1300 Morris Pk Ave, Bronx, NY 10461, USA. Molecular classification of head and neck squamous cell carcinoma using cDNA microarrays. *Cancer Res* **62:** (4) 1184.

Bosetti F, Seemann R, Bell JM, Zahorchak R, Friedman E, Rapoport SI, Manickam P. 2002. NIH/NIA, Brain Physiol & Metab Sect, 9000 Rockville Pike, Bldg 10, Room 6N202, Bethesda, Md 20892, USA. Analysis of gene expression with cDNA microarrays in rat brain after 7 and 42 days of oral lithium administration. *Brain Res Bull* **57:** (2) 205.

Chauhan D, Auclair D, Robinson EK, Hideshima T, Li GL, Podar K, Gupta D, Richardson P, Schlossman RL, Krett N, Chen LB, Munshi NC, Anderson KC*. 2002. *Harvard Univ, Dana Farber Canc Inst, 44 Binney St, Boston, Ma 02115, USA. Identification of genes regulated by dexamethasone in multiple myeloma cells using oligonucleotide arrays. *Oncogene* **21:** (9) 1346.

Cristillo AD, Bierer BE*. 2002. *NIH/NHLBI, Lab Lymphocyte Biol, Bldg 10, 10 Ctr Dr, Bethesda, Md 20892, USA. Identification of novel targets of immunosuppressive agents by cDNA-based microarray analysis. *J Biol Chem* **277:** (6) 4465.

Dan S, Tsunoda T, Kitahara O, Yanagawa R, Zembutsu H, Katagiri T, Yamazaki K, Nakamura Y, Yamori T*. 2002. *Japanese Fdn Canc Res, Div Mol Pharmacol, Toshima ku, 1-37-1 Kami-Ikebukuro, Tokyo 170 8455, Japan. An integrated database of chemosensitivity to 55 anticancer drugs and gene expression profiles of 39 human cancer cell lines. *Cancer Res* **62:** (4) 1139.

De Wit NJW, Burtscher HJ, Weidle UH, Ruiter DJ, Van Muijen GNP. 2002. Univ Med Ctr St Radboud, Dept Pathol, POB 9101, NL-6500 HB Nijmegen, The Netherlands. Differentially expressed genes identified in human melanoma cell lines with different metastatic behaviour using high density oligonucleotide arrays. *Melanoma Res* **12:** (1) 57.

Dong Y, Ganther HE, Stewart C, Ip C*. 2002. *Roswell Pk Canc Inst, Dept Expt Pathol, Elm & Carlton St, Buffalo, NY 14263, USA. Identification of molecular targets associated with selenium-induced growth inhibition in human breast cells using cDNA microarrays. *Cancer Res* **62:** (3) 708.

Eyster KM, Boles AL, Brannian JD, Hansen KA. 2002. Univ Sth Dakota, Div Basic Biomed Sci, Vermillion, SD 57069, USA. DNA microarray analysis of gene expression markers of endometriosis. *Fertil Steril* **77:** (1) 38.

Fink L, Kohlhoff S, Stein MM, Hanze J, Weissmann N, Rose F, Akkayagil E, Manz D, Grimminger F, Seeger W, Bohle RM. 2002. Univ Giessen, Inst Pathol, Langhansstr 10, DE-35392 Giessen, Germany. cDNA array hybridization after laser-assisted microdissection from non-neoplastic tissue. *Am J Pathol* **160:** (1) 81.

Galon J, Franchimont D, Hiroi N, Frey G, Boettner A, Ehrhart-Bornstein M, O'Shea JJ, Chrousos GP, Bornstein SR. 2001. Ctr Rech Biomed Cordeliers, INSERM U255, Lab Immunol Cellulaire & Clin, 15 rue Ecole Med, FR-75270 Paris 06, France. Gene profiling reveals unknown enhancing and suppressive actions of glucocorticoids on immune cells. *FASEB J* **16:** (1) 61.

Goldenberg D, Ayesh S, Schneider T, Pappo O, Jurim O, Eid A, Fellig Y, Dadon T, Ariel I, De Groot N, Hochberg A, Galun E. 2002. Hadassah Univ Hosp, Goldyne Savad Inst Gene Therapy, IL-91120 Jerusalem, Israel. Analysis of differentially expressed genes in hepatocellular carcinoma using cDNA arrays. *Mol Carcinog* **33:** (2) 113.

Hanash S, Brichory F, Beer D. 2001. Univ Michigan, Dept Pediat, 1150 West Med Ctr Dr, Ann Arbor, Mi 48109, USA. A proteomic approach to the identification of lung cancer markers. *Dis Marker* **17:** (4) 295.

Hippo Y, Taniguchi H, Tsutsumi S, Machida N, Chong JM, Fukayama M, Kodama T, Aburtani H*. 2002. *Univ Tokyo, Genome Sci Div, Meguro ku, 4-6-1 Komaba, Tokyo 153 8904, Japan. Global gene expression analysis of gastric cancer by oligonucleotide microarrays. *Cancer Res* **62:** (1) 233.

Hofmann WK, De Vos S, Elashoff D, Gschaidmeier H, Hoelzer D, Koeffler HP, Ottmann OG. 2002. Univ Hosp, Dept Haematol & Oncol, Theodor Stern Kai 7, DE-60596 Frankfurt, Germany. Relation between resistance of Philadelphia-chromosome-positive acute lymphoblastic leukaemia to the tyrosine kinase inhibitor ST1571 and gene-expression profiles: A gene-expression study. *Lancet* **359:** (9305) 481.

Hui ABY, Lo KW, Teo PML, To KF, Huang DP. 2002. Chinese Univ, Prince of Wales Hosp, Dept Anat & Cellular Pathol, Shatin, Hong Kong, Peoples Rep China. Genome wide detection of oncogene amplifications in nasopharyngeal carcinoma by array based comparative genomic hybridization. *Int J Oncol* **20:** (3) 467.

Leerkes MR, Caballero OL, Mackay A, Torloni H, O'Hare MJ, Simpson AJG, De Souza SJ*. 2002. *Ludwig Inst Canc Res, Rua Prof Antonio Prudente 109, BR-01509-010 Sao Paulo, Brazil. *In silico*-comparison of the transcriptome derived from purified normal breast cells and breast tumor cell lines reveals candidate upregulated genes in breast tumor cells. *Genomics* **79:** (2) 257.

Luo JH, Yu YP, Cieply K, Lin F, Deflavia P, Dhir R, Finkelstein S, Michalopoulos G, Becich M. 2002. Univ Pittsburgh, Dept Pathol, 3550 Terrace St, Pittsburgh, Pa 15261, USA. Gene expression analysis of prostate cancers. *Mol Carcinog* **33:** (1) 25.

Miller JC, Butler EB, Teh BS, Haab BB*. 2001. *Van Andel Res Inst, 333 Bostwick NE, Grand Rapids, Mi 49503, USA. The application of protein microarrays to serum diagnostics: Prostate cancer as a test case. *Dis Marker* **17:** (4) 225.

Moore SM, Nelson PS*. 2002. *Fred Hutchinson Canc Res Ctr, Div Human Biol, 1100 Fairview Ave, Nth Seattle, Wa 98109, USA. Gene expression profiling of the human prostate androgen response program. *J Androl* **23:** (2) 163.

Mori M, Mimori K, Yoshikawa Y, Shibuta K, Utsunomiya T, Sadanaga F, Tanaka F, Matsuyama A, Inoue H, Sugimachi K. 2002. Kyushu Univ, Med Inst Bioregulat, Beppu, Oita 874 0838, Japan. Analysis of the gene-expression profile regarding the progression of human gastric carcinoma. *Surgery* **131:** (1 Suppl) S39.

Noel-Georis I, Bernard A, Falmagne P, Wattiez R*. 2001. *Univ Mons, Lab Chim Biol, Ave Champ Mars 6, BE-7000 Mons, Belgium. Proteomics as the tool to search for lung disease markers in bronchoalveolar lavage. *Dis Marker* **17:** (4) 271.

Paweletz CP, Trock B, Pennanen M, Tsangaris T, Magnant C, Liotta LA, Petricoin EF*. 2001. *CBER/FDA, Tissue Proteomics Unit, Bethesda, Md 20892, USA. Proteomic patterns of nipple aspirate fluids obtained by SELDI-TOF: Potential for new biomarkers to aid in the diagnosis of breast cancer. *Dis Marker* **17:** (4) 301.

Petricoin EF, Ardekani AM, Hitt BA, Levine PJ, Fusaro VA, Steinberg SM, Mills GB, Simone C, Fishman DA, Kohn EC, Liotta LA. 2002. FDA/NIH Clin Proteomic Prog, Bldg 29A, Room 2B02, 8800 Rockville Pike, Bethesda, Md 20892, USA. Use of proteomic patterns in serum to identify ovarian cancer. *Lancet* **359:** (9306) 572.

Schubert EL, Hsu L, Cousens LA, Glogovac J, Self S, Reid BJ, Rabinovitch PS, Porter PL*. 2002. *Univ Washington, Dept Pathol, 1959 NW Pacific St, Seattle, Wa 98195, USA. Single nucleotide polymorphism array analysis of flow-sorted epithelial cells from frozen *versus* fixed tissues for whole genome analysis of allelic loss in breast cancer. *Am J Pathol* **160:** (1) 73.

Selaru FM, Zou T, Xu Y, Shustova V, Yin J, Mori Y, Sato F, Wang S, Olaru A, Shibata D, Greenwald BD, Krasna MJ, Abraham JM, Meltzer SJ*. 2002. *Univ Maryland, 22 Sth Greene St, Baltimore, Md 21201, USA. Global gene expression profiling in Barrett's esophagus and esophageal cancer: A comparative analysis using cDNA microarrays. *Oncogene* **21:** (3) 475.

Shalhoub P, Kern S, Girard S, Beretta L*. 2001. *Univ Michigan, Dept Microbiol & Immunol, 1150 West Med Ctr Dr, Ann Arbor, Mi 48109, USA. Proteomic-based approach for the identification of tumor markers associated with hepatocellular carcinoma. *Dis Marker* **17:** (4) 217.

Tabuchi Y, Kondo T*, Ogawa R, Mori H. 2002. *Med & Pharmeceut Univ, Dept Radiol Sci, 2630 Sugitani, Toyama 930 0194, Japan. DNA microarray analyses of genes elicited by ultrasound in human U937 cells. *Biochem Biophys Res Commun* **290:** (1) 498.

Tannapfel A, Geissler F, Witzigmann H, Hauss J, Wittekind C. 2001.

Copyright © 2002 John Wiley & Sons, Ltd. 

Univ Leipzig, Inst Pathol, Liebigstr 26, DE-04103 Leipzig, Germany. Analysis of liver allograft rejection related genes using cDNA-microarrays in liver allograft specimen. *Transpl Proc* **33:** (7-8) 3283.

Ten Hagen KG, Balys MM, Tabak LA, Melvin JE*. 2002. *Univ Rochester, Ctr Oral Biol, 601 Elmwood Ave, Rochester, NY 14642, USA. Analysis of isoproterenol-induced changes in parotid gland gene expression. *Physiol Genomics* **8:** (2) 107.

Tsuji T, Shimohama S*. 2001. *Kyoto Univ, Dept Neurol, Sankyo ku, 54 Shogoin Kawaharacho, Kyoto 606, Japan. Analysis of the proteomic profiling of brain tissue in Alzheimer's disease. *Dis Marker* **17:** (4) 247.

Wai DH, Schaefer KL, Schramm A, Korsching E, Van Valen F, Ozaki T, Boecker W, Schweigerer L, Dockhorn-Dworniczak B, Poremba C*. 2002. *Univ Munster, Domagk Inst Pathol, Domagkstr 17, DE-48149 Munster, Germany. Expression analysis of pediatric solid tumor cell lines using oligonucleotide microarrays. *Int J Oncol* **20:** (3) 441.

Zembutsu H, Ohnishi Y, Tsunoda T, Furukawa Y, Katagiri T, Ueyama Y, Tamaoki N, Nomura T, Kitahara O, Yanagawa R, Hirata K, Nakamura Y*. 2002. *Univ Tokyo, Ctr Human Genome, Minato ku, 4-6-1 Shirokanedai, Tokyo 108 8639, Japan. Genome-wide cDNA microarray screening to correlate gene expression profiles with sensitivity of 85 human cancer xenografts to anticancer drugs. *Cancer Res* **62:** (2) 518.

### 8 EST, cDNA and other clone resources

Carson DL, Huckett BI, Botha FC. 2002. SA Sugar Assoc Expt Stn, Biotechnol Dept, Priv Bag X02, ZA-4300 Mt Edgecombe, Rep Sth Africa. Sugarcane ESTs differentially expressed in immature and maturing internodal tissue. *Plant Sci* **162:** (2) 289.

Jia L, Young MF, Powell J, Yang LM, Ho NC, Hotchkiss R, Robey PG, Francomano CA*. 2002. *NIH/NIA, Genet Lab, Baltimore, Md 21224, USA. Gene expression profile of human marrow stromal cells: High-throughput expressed sequence tag sequencing analysis. *Genomics* **79:** (1) 7.

Michalek W, Weschke W, Pleissner KP, Graner A. 2002. Planta GmbH, Grimsehlstr 31, DE-37555 Einbeck, Germany. EST analysis in barley defines a unigene set comprising 4,000 genes. *Theor Appl Genet* **104:** (1) 97.

Yao J, Burton JL, Saama P, Sipkovsky S, Coussens PM*. 2001. *Michigan State Univ, Dept Anim Sci, Anthony Hall, East Lansing, Mi 48824, USA. Generation of EST and cDNA microarray resources for the study of bovine immunobiology. *Acta Vet Scand* **42:** (3) 391.

### 9 Functional genomics

Avaro S, Belgareh-Touze N, Sibella-Arguelles C, Volland C, Haguenauer-Tsapis R*. 2002. *Univ Paris 6 & Paris 7, Inst Jacques Monod-CNRS, 2 Pl Jussieu, FR-75251 Paris 05, France. Mutants defective in secretory/vacuolar pathways in the EUROFAN collection of yeast disruptants. *Yeast* **19:** (4) 351.

Briza P, Bogengruber E, Thur A, Rutzler M, Munsterkotter M, Dawes IW, Breitenbach M*. 2002. *Univ Salzburg, Inst Genet & Allgemeine Biol, Helbrunnerstr 34, AU-5020 Salzburg, Austria. Systematic analysis of sporulation phenotypes in 624 non-lethal homozygous deletion strains of *Saccharomyces cerevisiae*. *Yeast* **19:** (5) 403.

Cujec TP, Medeiros PF, Hammond P, Rise C, Kreider BL. 2002. Phylos, 128 Spring St, Lexington, Ma 02421, USA. Selection of v-abl tyrosine kinase substrate sequences from randomized peptide and cellular proteomic libraries using mRNA display. *Chem Biol* **9:** (2) 253.

Featherstone DE, Broadie K. 2002. Univ Utah, Dept Biol, 257 Sth 1400 East, Salt Lake City, Ut 84112, USA. Wrestling with pleiotropy: Genomic and topological analysis of the yeast gene expression network. *Bioessays* **24:** (3) 267.

Higgins VJ, Alic N, Thorpe GW, Breitenbach M, Larsson V, Dawes IW. 2002. Univ NSW, Sch Biochem & Molec Genet, Sydney, NSW 2052, Australia. Phenotypic analysis of gene deletant strains for sensitivity to oxidative stress. *Yeast* **19:** (3) 203.

Klevecz RR, Murray DB. 2001. City of Hope Natl Med Ctr, Dept Biol, Duarte, Ca 91010, USA. Genome wide oscillations in expression: Wavelet analysis of time series data from yeast expression arrays uncovers the dynamic architecture of phenotype. *Mol Biol Rep* **28:** (2) 73.

Marra A, Asundi J, Bartilson M, Lawson S, Fang F, Christine J, Wiesner C, Brigham D, Schneider WP, Hromockyj AE. 2002. Pfizer Global R&D, Antibacterials Discovery, Eastern Point Rd, Groton, Ct 06340, USA. Differential fluorescence induction analysis of *Streptococcus pneumoniae* identifies genes involved in pathogenesis. *Infect Immun* **70:** (3) 1422.

Reid RJD, Sunjevaric I, Kedacche M, Rothstein R*. 2002. *Columbia Univ, Coll Phys & Surg, Dept Genet & Dev, New York, NY 10032, USA. Efficient PCR-based gene disruption in *Saccharomyces* strains using intergenic primers. *Yeast* **19:** (4) 319.

Wooff E, Michell SL, Gordon SV, Chambers MA, Bardarov S, Jacobs WR, Hewinson RG, Wheeler PR*. 2002. *Vet Labs Agcy, TB Res Grp, Weybridge, England. Functional genomics reveals the sole sulphate transporter of the *Mycobacterium tuberculosis* complex and its relevance to the acquisition of sulphur *in vivo*. *Mol Microbiol* **43:** (3) 653.

### 10 Transcriptomics

Becerra M, Lombardia-Ferreira LJ, Hauser NC, Hoheisel JD, Tizon B, Cerdan ME. 2002. Univ La Coruna, Dept Biol Cellular & Mol, Campus La Zapateira s/n, ES-15075 La Coruna, Spain. The yeast transcriptome in aerobic and hypoxic conditions: Effects of *hap1, rox1, rox3* and *srb10* deletions. *Mol Microbiol* **43:** (3) 545.

Choi EJ, Ha CM, Choi JG, Kang SS, Choi WS, Park SK, Kim KJ, Lee BJ*. 2001. *Univ Ulsan, Dept Biol Sci, Ulsan 680749, South Korea. Low-density cDNA array-coupled to PCR differential display identifies new estrogen-responsive genes during the postnatal differentiation of the rat hypothalamus. *Mol Brain Res* **97:** (2) 115.

Eaves IA, Wicker LS, Ghandour G, Lyons PA, Peterson LB, Todd JA*, Glynne RJ. 2002. *Univ Cambridge, Addenbrookes Hosp, Cambridge Inst Med Res, Cambridge CB2 2XY, England. Combining mouse congenic strains and microarray gene expression analyses to study a complex trait: The NOD model of type 1 diabetes. *Genome Res* **12:** (2) 232.

Fan M, Mi RF, Yew DT, Chan WY*. 2001. *Chinese Univ Hong Kong, Dept Anat, Shatin, Hong Kong, Peoples Rep China. Analysis of gene expression following sciatic nerve crush and spinal cord hemisection in the mouse by microarray expression profiling. *Cell Mol Neurobiol* **21:** (5) 497.

Ivashuta S, Uchiyama K, Gau M, Shimamoto Y. 2002. Natl Agr Res Ctr, Hitsujigaoka 1, Sapporo, Hokkaido 062 855, Japan. Linear amplification coupled with controlled extension as a means of probe amplification in a cDNA array and gene expression analysis during cold acclimation in alfalfa (*Medicago sativa* L.). *J Exp Bot* **53:** (367) 351.

Jarmer H, Berka R, Knudsen S, Saxild HH*. 2002. *TU Denmark, Biocentrum, DK-2800 Lyngby, Denmark. Transcriptome analysis documents induced competence of *Bacillus subtilis* during nitrogen limiting conditions. *FEMS Microbiol Lett* **206:** (2) 197.

Keyvani K, Witte OW, Paulus W. 2002. Univ Hosp, Inst Neuropathol, Domagkstr 19, DE-48129 Munster, Germany. Gene expression profiling in perilesional and contralateral areas after ischemia in rat brain. *J Cereb Blood Flow Metabol* **22:** (2) 153.

Lehner B, Williams G, Campbell RD, Sanderson CM*. 2002. *MRC UK HGMP Resource Ctr, Cambridge CB10 1SB, England. Antisense transcripts in the human genome. *Trends Genet* **18:** (2) 63.

Marc P, Margeot A, Devaux F, Blugeon C, Corral-Debrinski M, Jacq C*. 2002. *Ecole Normale Super, CNRS UMR 8541, Lab Mol Genet, 46 rue Ulm, FR-75005 Paris, France. Genome-wide analysis of mRNAs targeted to yeast mitochondria. *EMBO Rep* **3:** (2) 159.

Matsuyama T, Tamaoki M, Nakajima N, Aono M, Kubo A, Moriya S, Ichihara T, Suzuki O, Saji H*. 2002. *Natl Inst Environm Studies, Div Environm Biol, 16-2 Onogawa, Tsukuba, Ibaraki 305 0053, Japan. cDNA microarray assessment for ozone-stressed *Arabidopsis thaliana*. *Environ Pollut* **117:** (2) 191.

Paulino LC, De Mello MP, Ottoboni LMM*. 2002. *UNICAMP, CBMEG, CP 6010, BR-13083-970 Campinas, SP, Brazil. Differential gene expression in response to copper in *Acidithiobacillus ferroxidans* analyzed by RNA arbitrarily primed polymerase chain reaction. *Electrophoresis* **23:** (4) 520.

Salmelin C, Vilpo J*. 2002. *Tampere Univ Hosp, Dept Clin Chem, FI-33521 Tampere, Finland. Expression profiling of all protein-coding genes in wild-type and three DNA repair-deficient substrains of *Escherichia coli* K-12. *Comp Funct Genom* **3:** (1) 3.

Copyright © 2002 John Wiley & Sons, Ltd. 

Serrano EE, Trujillo-Provencio C, Sultemeier DR, Bullock WM, Quick QA. 2001. NM State Univ, Dept Biol, Las Cruces, NM 88003, USA. Identification of genes expressed in the *Xenopus* inner ear. *Cell Mol Biol (Noisy-le-Grand)* **47:** (7) 1229.

Seth P, Krop I, Porter D, Polyak K*. 2002. *Dana Farber Canc Inst, Dept Adult Oncol, 44 Binney St, Boston, Ma 02115, USA. Novel estrogen and tamoxifen induced genes identified by SAGE (Serial Analysis of Gene Expression). *Oncogene* **21:** (5) 836.

Sreenivasulu N, Altschmied L, Panitz R, Hahnel U, Michalek W, Weschke W, Wobus U*. 2002. *IPK, Corrensstr 3, DE-06466 Gatersleben, Germany. Identification of genes specifically expressed in maternal and filial tissues of barley caryopses: A cDNA array analysis. *Mol Genet Genomics* **266:** (5) 758.

Teng SC, Epstein C, Tsai YL, Cheng HW, Chen HL, Lin JJ. 2002. Natl Taiwan Univ, Dept Microbiol, 1 Sec 1 Jan Ai Rd, Taipei 10018, Taiwan. Induction of global stress response in *Saccharomyces cerevisiae* cells lacking telomerase. *Biochem Biophys Res Commun* **291:** (3) 714.

Thompson DK, Beliaev AS, Giometti CS, Tollaksen SL, Khare T, Lies DP, Nealson KH, Lim J, Yates J, Brandt CC, Tiedje JM, Zhou J*. 2002. *Oak Ridge Natl Lab, Div Environm Sci, Oak Ridge, Tn 37831, USA. Transcriptional and proteomic analysis of a ferric uptake regulator (fur) mutant of *Shewanella oneidensis*: Possible involvement of fur in energy metabolism, transcriptional regulation, and oxidative stress. *Appl Environ Microbiol* **68:** (2) 881.

### 11 Proteomics

Ahram M, Best CJM, Flaig MJ, Gillespie JW, Leiva IM, Chuaqui RF, Zhou G, Shu HJ, Duray PH, Linehan WM, Raffeld M, Ornstein DK, Zhao Y, Petricoin EF, Emmert-Buck MR*. 2002. *NIH/NCI, Pathogenet Unit, 10 Ctr Dr, Bldg 10, Bethesda, Md 20892, USA. Proteomic analysis of human prostate cancer. *Mol Carcinog* **33:** (1) 9.

Andersen JS, Lyon CE, Fox AH, Leung AKL, Lam YW, Steen H, Mann M, Lamond AI*. 2002. *Univ Sthn Denmark, Dept Biochem & Mol Biol, Campusvej 55, DK-5230 Odense M, Denmark. Directed proteomic analysis of the human nucleolus. *Curr Biol* **12:** (1) 1.

Antonucci F, Chilosi M, Santacatterina M, Herbert B, Righetti PG*. 2002. *Univ Verona, Dept Agr & Ind Biotechnol, Strada Grazie 15, IT-37134 Verona, Italy. Proteomics and immunomapping of reactive lymph-node and lymphoma. *Electrophoresis* **23:** (2) 356.

Beranova-Giorgianni S, Pabst MJ, Russell TM, Giorgianni F, Goldowitz D, Desiderio DM*. 2002. *Univ Tennessee, Stout Neurosci Mass Spectrometry Lab, 847 Monroe Ave, Memphis, Tn 38163, USA. Preliminary analysis of the mouse cerebellum proteome. *Mol Brain Res* **98:** (1-2) 135.

Castagna A, Campostrini N, Farinazzo A, Zanusso G, Monaco S, Righetti PG*. 2002. *Univ Verona, Dept Agr & Ind Biotechnol, Strada Grazie 15, IT-37134 Verona, Italy. Comparative two-dimensional mapping of prion protein isoforms in human cerebrospinal fluid and central nervous system. *Electrophoresis* **23:** (2) 339.

Charlwood J, Hanrahan S, Tyldesley R, Langridge J, Dwek M, Camilleri P*. 2002. *GlaxoSmithKline, New Frontiers Sci Pk, 3rd Ave, Harlow CM19 5AW, England. Use of proteomic methodology for the characterization of human milk fat globular membrane proteins. *Anal Biochem* **301:** (2) 314.

Cohen AM, Rumpel K, Coombs GH, Wastling JM*. 2002. *Univ Glasgow, Inst Biomed & Life Sci, Joseph Black Bldg, Glasgow G12 8QQ, Scotland. Characterisation of global protein expression by two-dimensional electrophoresis and mass spectrometry: Proteomics of *Toxoplasma gondii*. *Int J Parasitol* **32:** (1) 39.

Ficarro SB, McCleland ML, Stukenberg PT, Burke DJ, Ross MM, Shabanowitz J, Hunt DF, White FM*. 2002. *MDS Proteomics, Charlottesville, Va 22901, USA. Phosphoproteome analysis by mass spectrometry and its application to *Saccharomyces cerevisiae*. *Nat Biotechnol* **20:** (3) 301.

Fountoulakis M, Berndt P, Langen H, Suter L. 2002. F Hoffmann La Roche & Co Ltd, Genomics Technol, CH-4070 Basel, Switzerland. The rat liver mitochondrial proteins. *Electrophoresis* **23:** (2) 311.

Harris RA, Yang A, Stein RC, Lucy K, Brusten L, Herath A, Parekh R, Waterfield MD, O'Hare MJ, Neville MA, Page MJ, Zvelebil MJ*. 2002. *Ludwig Inst Canc Res, 91 Riding Hse St, London W1 7BS, England. Cluster analysis of an extensive human breast cancer cell line protein expression map database. *Proteomics* **2:** (2) 212.

Harrison PM, Kumar A, Lang N, Snyder M, Gerstein M*. 2002. *Yale Univ, Dept Mol Biophys & Biochem, 266 Whitney Ave, New Haven, Ct 06520, USA. A question of size: The eukaryotic proteome and the problems in defining it. *Nucleic Acids Res* **30:** (5) 1083.

Karlin S, Brocchieri L, Bergman A, Mrazek J, Gentles AJ. 2002. Stanford Univ, Dept Math, Stanford, Ca 94305, USA. Amino acid runs in eukaryotic proteomes and disease associations. *Proc Natl Acad Sci U S A* **99:** (1) 333.

Karlsen AE, Sparre T, Nielsen K, Nerup J, Pociot F*. 2001. *Steno Diabet Ctr, Niels Steensensvej 2, DK-2820 Gentofte, Denmark. Proteome analysis: A novel approach to understand the pathogenesis of type 1 diabetes mellitus. *Dis Marker* **17:** (4) 205.

Kim SI, Kim SJ, Nam MH, Kim SH, Ha KS, Oh KH, Yoo JS, Park YM. 2002. Korea Basic Sci Inst, Biomol Res Team, Taejon 305333, South Korea. Proteome analysis of aniline-induced proteins in *Acinetobacter lwoffii* K24. *Curr Microbiol* **44:** (1) 61.

Maltman DJ, Simon WJ, Wheeler CH, Dunn MJ, Wait R, Slabas AR*. 2002. *Univ Durham, Dept Biol Sci, Durham DH1 3LE, England. Proteomic analysis of the endoplasmic reticulum from developing and germinating seed of castor (*Ricinus communis*). *Electrophoresis* **23:** (4) 626.

Martin SAM, Cash P, Blaney S, Houlihan DF. 2001. Univ Aberdeen, Dept Zool, Aberdeen AB24 2TZ, Scotland. Proteome analysis of rainbow trout (*Oncorhynchus mykiss*) liver proteins during short term starvation. *Fish Physiol Biochem* **24:** (3) 259.

Musante L, Candiano C, Bruschi M, Zennaro C, Carraro M, Artero M, Giuffrida MG, Conti A, Santucci A, Ghiggeri GM*. 2002. *Ist Giannina Gaslini, Lab Fisiopatol Uremia, Largo G Gaslini 5, IT-16148 Genda, Italy. Characterization of plasma factors that alter the permeability to albumin within isolated glomeruli. *Proteomics* **2:** (2) 197.

Park SG, Kho CW, Cho S, Lee DH, Kim SH, Park BC*. 2002. *KRIBB, Proteome Res Lab, POB 115, Taejon 305600, South Korea. A functional proteomic analysis of secreted fibrinolytic enzymes from *Bacillus subtilis* 168 using a combined method of two-dimensional gel electrophoresis and zymography. *Proteomics* **2:** (2) 206.

Peltier JB, Emanuelsson O, Kalume DE, Ytterberg J, Friso G, Rudella A, Liberles DA, Soderberg L, Roepstorff P, Von Heijne G, Van Wijk KJ*. 2002. *Cornell Univ, Dept Plant Biol, Ithaca, NY 14853, USA. Central functions of the lumenal and peripheral thylakoid proteome of *Arabidopsis* determined by experimentation and genome-wide prediction. *Plant Cell* **14:** (1) 211.

Piubelli C, Galvani M, Hamdan M, Domenici E, Righetti PG*. 2002. *Univ Verona, Dept Agr & Ind Biotechnol, Strada Grazie 15, IT-37134 Verona, Italy. Proteome analysis of rat polymorphonuclear leukocytes: A two-dimensional electrophoresis/mass spectrometry approach. *Electrophoresis* **23:** (2) 298.

Rabilloud T, Strub JM, Carte N, Luche S, Van Dorsselaer A, Lunardi J, Giege R, Florentz C*. 2002. *Inst Biol Mol & Cellulaire, CNRS UPR 9002, 15 rue Descartes, FR-67084 Strasbourg, France. Comparative proteomics as a new tool for exploring human mitochondrial tRNA disorders. *Biochemistry* **41:** (1) 144.

Roos C, Kolmer M, Mattila P, Renkonen R*. 2002. *Univ Helsinki, Rational Drug Design Prog, Biomedicum, FI-00014 Helsinki, Finland. Composition of *Drosophila melanogaster* proteome involved in fucosylated glycan metabolism. *J Biol Chem* **277:** (5) 3168.

Rosen R, Buttner K, Becher D, Nakahigashi K, Yura T, Hecker M, Ron EZ*. 2002. *Tel Aviv Univ, Dept Mol Microbiol & Biotechnol, IL-69978 Tel Aviv, Israel. Heat shock proteome of *Agrobacterium tumefaciens*: Evidence for new control systems. *J Bacteriol* **184:** (6) 1772.

Schmidt F, Lueking A, Nordhoff E, Gobom J, Klose J, Seitz H, Egelhofer V, Eickhoff H, Lehrach H, Cahill DJ*. 2002. *Max-Planck-Inst Mol Genet, Ihnestr 73, DE-14195 Berlin, Germany. Generation of minimal protein identifiers of proteins from two-dimensional gels and recombinant proteins. *Electrophoresis* **23:** (4) 621.

Shaw AC, Gevaert K, Demol H, Hoorelbeke B, Vandekerckhove J, Larsen MR, Roepstorff P, Holm A, Christiansen G, Birkelund S. 2002. Univ Aarhus, Dept Med Microbiol & Immunol, Bartholin Bldg, DK-8000 Aarhus C, Denmark. Comparative proteome analysis of *Chlamydia trachomatis* serovar A, D and L2. *Proteomics* **2:** (2) 164.

Sherman NE, Stefansson B, Fox JW, Goldberg JB*. 2001. *Univ Virginia, Dept Microbiol, Charlottesville, Va 22908, USA. *Pseudomonas aeruginosa* and a proteomic approach to bacterial pathogenesis. *Dis Marker* **17:** (4) 285.

Van Loo G, Demol H, Van Gurp M, Hoorelbeke B, Schotte P, Beyaert

Copyright © 2002 John Wiley & Sons, Ltd. 

R, Zhivotovsky B, Gevaert K, Declercq W, Vanderkerckhove J, Vandenabeele P*. 2002. *Flanders Interuniv, Inst Biotechnol, Kl Ledeganckstr 35, BE-9000 Ghent, Belgium. A matrix-assisted laser desorption/ionization post-source decay (MALDI-PSD) analysis of proteins released from isolated liver mitochondria treated with recombinant truncated Bid. *Cell Death Differ* **9:** (3) 301.

Wang YY, Khoo KH, Chen ST, Lin CC, Wong CH*, Lin CH. 2002. *Scripps Res Inst, La Jolla, Ca 92037, USA. Studies on the immunomodulating and antitumor activities of *Ganoderma lucidum* (Reishi) polysaccharides: Functional and proteomic analyses of a fucose-containing glycoprotein fraction responsible for the activities. *Bioorg Med Chem* **10:** (4) 1057.

Werhahn W, Braun HP*. 2002. *Univ Hannover, Inst Angew Genet, Herrenhauser Str 2, DE-30419 Hannover, Germany. Biochemical dissection of the mitochondrial proteome from *Arabidopsis thaliana* by three-dimensional gel electrophoresis. *Electrophoresis* **23:** (4) 640.

Woo SH, Fukuda M, Islam N, Takaoka M, Kawasaki H, Hirano H*. 2002. *Yokohama City Univ, Kihara Inst Biol Res, Maioka 641 12, Yokohama, Kanagawa, Japan. Efficient peptide mapping and its application to identify embryo proteins in rice proteome analysis. *Electrophoresis* **23:** (4) 647.

Yee A, Chang XQ, Pineda-Lucena A, Wu B, Semesi A, Le B, Ramelot T, Lee GM, Bhattacharyya S, Gutierrez P *et al*. 2002. c/o Arrowsmith CH, Univ Toronto, Ontario Canc Inst, 101 Coll St, Toronto, Ontario, Canada M5G 1L7. An NMR approach to structural proteomics. *Proc Natl Acad Sci U S A* **99:** (4) 1825.

### 13 Metabolomics

Schuster S, Pfeiffer T, Moldenhauer F, Koch I, Dandekar T. 2002. Max Delbruck Ctr Mol Med, Dept Bioinformatics, DE-13092 Berlin, Germany. Exploring the pathway structure of metabolism: Decomposition into subnetworks and application to *Mycoplasma pneumoniae*. *Bioinformatics* **18:** (2) 351.

### 14 Genomic approaches to development

Cho KS, Choi JG, Ha CM, Son YJ, Choi WS, Lee BJ*. 2002. *Univ Ulsan, Dept Biol Sci, Ulsan 680749, South Korea. Comparison of gene expression in old *versus* young rat hippocampus by cDNA array. *Neuroreport* **13:** (3) 285.

Doi M, Nagano A, Nakamura Y*. 2002. * Univ Tokyo, Ctr Human Genome, Minato ku, 4-6-1 Shirokanedai, Tokyo 108 8639, Japan. Genome-wide screening by cDNA microarray of genes associated with matrix mineralization by human mesenchymal stem cells *in vitro*. *Biochem Biophys Res Commun* **290:** (1) 381.

Han SH, McCool BA, Murchison D, Nahm SS, Parrish AR, Griffith WH*. 2002. *Texas A&M Univ, Syst Hlth Sci Ctr, Dept Med Pharmacol & Toxicol, College Station, Tx 77843, USA. Single-cell RT-PCR detects shifts in mRNA expression profiles of basal forebrain neurons during aging. *Mol Brain Res* **98:** (1-2) 67.

Izzo MW, Pucci B, Tuan RS, Hall DJ*. 2002. *Thomas Jefferson Univ, Dept Orthopaed Surg, 1015 Walnut St, Philadelphia, Pa 19107, USA. Gene expression profiling following BMP-2 induction of mesenchymal chondrogenesis *in vitro*. *Osteoarthritis Cartilage* **10:** (1) 23.

Kusakabe T, Yoshida R, Kawakami I, Kusakabe R, Mochizuki Y, Yamada L, Shini T, Kohara Y, Satoh N, Tsuda M, Satou Y. 2002. Himeji Inst Technol, Dept Life Sci, 3-2-1 Kouto, Hyogo 678 1297, Japan. Gene expression profiles in tadpole larvae of *Ciona intestinalis*. *Dev Biol* **242:** (2) 188.

Le Q, Soprano DR, Soprano KJ*. 2002. *Temple Univ, Dept Microbiol & Immunol, 3400 Nth Broad St, Philadelphia, Pa 19140, USA. Profiling of retinoid mediated gene expression in synchronized human SCC cells using Atlas™ human cDNA expression arrays. *J Cell Physiol* **190:** (3) 345.

Saito S, Matoba R, Ueno N, Matsubara K, Kato K*. 2002. *Inst Sci & Technol, Taisho Lab Funct Genomics, 8916-5 Takayama, Nara 630 0101, Japan. Comparison of gene expression profiling during postnatal development of mouse dentate gyrus and cerebellum. *Physiol Genomics* **8:** (2) 131.

Stokes DG, Liu G, Coimbra IB, Piera-Velazquez S, Crowl RM, Jimenez SA*. 2002. *Thomas Jefferson Univ, Dept Med, 233 Sth 10th St, Philadelphia, Pa 19107, USA. Assessment of the gene expression profile of differentiated and dedifferentiated human fetal chondrocytes by microarray analysis. *Arthritis Rheum* **46:** (2) 404.

### 15 Technological advances

Avseenko NV, Morozova TY, Ataullakhanov FI, Morozov VN*. 2002. *Russian Acad Sci, Inst Theoret & Expt Biophys, RU-142290 Pushchino, Russia. Immunoassay with multicomponent protein microarrays fabricated by electrospray deposition. *Anal Chem* **74:** (5) 927.

Beier M, Hoheisel JD*. 2002. *Deutsch Krebsforschungszentrum, Funct Genome Anal, Neuenheimer Feld 506, DE-69120 Heidelberg, Germany. Analysis of DNA-microarrays produced by inverse *in situ* oligonucleotide synthesis. *J Biotechnol* **94:** (1) 15.

Brown VM, Ossadtchi A, Khan AH, Cherry SR, Leahy RM, Smith AJ*. 2002. *UCLA, Dept Mol & Med Pharmacol, Los Angeles, Ca 90095, USA. High-throughput imaging of brain gene expression. *Genome Res* **12:** (2) 244.

Brown VM, Ossadtchi A, Khan AH, Gambhir SS, Cherry SR, Leahy RM, Smith DJ*. 2002. *Address as above. Gene expression tomography. *Physiol Genomics* **8:** (2) 159.

Candiano G, Musante L, Bruschi M, Ghiggeri GM, Herbert B, Antonucci F, Righetti PG*. 2002. *Univ Verona, Dept Agr & Ind Biotechnol, Strada Grazie 15, IT-37134 Verona, Italy. Two-dimensional maps in soft immobilized pH gradient gels: A new approach to the proteome of the Third Millennium. *Electrophoresis* **23:** (2) 292.

Graves DJ, Su HJ, Addya S, Surrey S, Fortina P. 2002. Univ Penn, Sch Engn & Appl Sci, 220 Sth 33 St, Philadelphia, Pa 19104, USA. Four-laser scanning confocal system for microarray analysis. *Biotechniques* **32:** (2) 346.

Hamadeh HK, Bushel P, Tucker CJ, Martin K, Paules R, Afshari CA*. 2002. *Nat Inst Environ Hlth Sci, POB 12233, Alexander Dr, Res Triangle Park, NC 27709, USA. Detection of diluted gene expression alterations using cDNA microarrays. *Biotechniques* **32:** (2) 322.

Holter JL, Humphries A, Crunelli V, Carter DA*. 2001. *Univ Wales Coll Cardiff, Sch Biosci, Cardiff CF10 3US, Wales. Optimisation of methods for selecting candidate genes from cDNA array screens: Application to rat brain punches and pineal. *J Neurosci Methods* **112:** (2) 173.

Hoving S, Gerrits B, Voshol H, Muller D, Roberts RC, Van Oostrum J. 2002. Novartis Pharma AG, Proteome Sci Unit, WSJ-88-801, CH-4002 Basel, Switzerland. Preparative two-dimensional gel electrophoresis at alkaline pH using narrow range immobilized pH gradients. *Proteomics* **2:** (2) 127.

Jain AN, Tokuyasu TA, Snijders AM, Segraves R, Albertson DG, Pinkel D. 2002. Univ Calif, Canc Ctr, Box 0128, San Francisco, Ca 94143, USA. Fully automatic quantification of microarray image data. *Genome Res* **12:** (2) 325.

Khatri P, Draghici S, Ostermeier GC, Krawetz SA*. 2002. *Wayne State Univ, Inst Comput Sci, Detroit, Mi 48201, USA. Profiling gene expression using Onto-Express. *Genomics* **79:** (2) 266.

Khodarev NN, Yu J, Nodzenski E, Murley JS, Kataoka Y, Brown CK, Grdina DJ, Weichselbaum RR*. 2002. *Univ Chicago, Dept Radiat & Cellular Oncol, 5841 Sth Maryland Ave, Chicago, Il 60637, USA. Method of RNA purification from endothelial cells for DNA array experiments. *Biotechniques* **32:** (2) 316.

Leimgruber RM, Malone JP, Radabaugh MR, La Porte ML, Violand BN, Monahan JB. 2002. Pharmacia Corp, Discovery R&D, 700 Chesterfield Pkwy Nth, St Louis, Mo 63198, USA. Development of improved cell lysis, solubilization and imaging approaches for proteomic analyses. *Proteomics* **2:** (2) 135.

Liang XW, Teng A, Braun DM, Felgner J, Wang Y, Baker SI, Chen SZ, Zelphati O, Felgner PL*. 2002. *Gene Therapy Syst Inc, 10190 Telesis Ct, San Diego, Ca 92121, USA. Transcriptionally active polymerase chain reaction (TAP): High throughput gene expression using genome sequence data. *J Biol Chem* **277:** (5) 3593.

Mayer C, Palkovits R, Bauer G, Schalkhammer T*. 2001. *Delft Univ Technol, Kluyver Lab Biotechnol, Julianalaan 67, NL-2628 BC Delft, The Netherlands. Surface enhanced resonance of metal nano clusters: A novel tool for proteomics. *J Nanopart Res* **3:** (5-6) 361.

Pluskal MG, Bogdanova A, Lopez M, Gutierrez S, Pitt AM. 2002. Proteome Syst, 14 Gill St, Woburn, Ma 01801, USA. Multiwell in-gel protein digestion and microscale sample preparation for protein identi-

Copyright © 2002 John Wiley & Sons, Ltd.

fication by mass spectrometry. *Proteomics* **2:** (2) 145.

Pratt JM, Robertson DHL, Gaskell SJ, Riba-Garcia I, Hubbard SJ, Sidhu K, Oliver SG, Butler P, Hayes A, Petty J, Beynon RJ*. 2002. *Univ Liverpool, Dept Preclin Vet Sci, Liverpool L69 7ZJ, England. Stable isotope labelling *in vivo* as an aid to protein identification in peptide mass fingerprinting. *Proteomics* **2:** (2) 157.

Ros A, Faupel M, Mees H, Van Oostrum J, Ferrigno R, Reymond F, Michel P, Rossier JS, Girault HH*. 2002. *Ecole Polytech Fed Lausanne, Lab Electrochim, CH-105 Lausanne, Switzerland. Protein purification by off-gel electrophoresis. *Proteomics* **2:** (2) 151.

Salin H, Vujasinovic T, Mazurie A, Maitrejean S, Menini C, Mallet J, Dumas S*. 2002. *Hop La Pitie Salpetriere, CNRS UMR 7091, LGN, 83 Blvd Hop, FR-75013 Paris, France. A novel sensitive microarray approach for differential screening using probes labelled with two different radioelements (Electronic paper). *Nucleic Acids Res* **30:** (4) E17.

### 16 Bioinformatics

Azuaje F. 2002. Univ Dublin - Trinity Coll, Dept Comp Sci, Dublin 2, Rep Ireland. A cluster validity framework for genome expression data. *Bioinformatics* **18:** (2) 319.

Begley DA, Ringwald M. 2002. Jackson Lab, 600 Main St, Bar Harbor, Me 04609, USA. Electronic tools to manage gene expression data. *Trends Genet* **18:** (2) 108.

Bouton CMLS, Pevsner J*. 2002. *Johns Hopkins Sch Med, Dept Neurosci, Baltimore, Md 21205, USA. DRAGON View: Information visualization for annotated microarray data. *Bioinformatics* **18:** (2) 323.

Brasier AR. 2002. Univ Texas, Div Endocrinol, 301 Univ Blvd, Galveston, Tx 77555, USA. Retriever and CompareTable, two informatics tools for data analysis of high-density oligonucleotide arrays. *Biotechniques* **32:** (1) 100.

Bumm K, Zheng MZ, Bailey C, Zhan FH, Chiriva-Internati M, Eddlemon P, Terry J, Barlogie B, Shaughnessy JD*. 2002. *Univ Arkansas Med Sci, Lambert Lab Myeloma Genet, Little Rock, Ar 72205, USA. CGO: Utilizing and integrating gene expression of microarray data in clinical research and data management. *Bioinformatics* **18:** (2) 327.

Cawley SE, Wirth AI, Speed TP. 2001. Univ Calif Berkeley, Dept Stat, Berkeley, Ca 94720, USA. Phat: A gene finding program for *Plasmodium falciparum*. *Mol Biochem Parasitol* **118:** (2) 167.

Cline M, Hughey R, Karplus K. 2002. Univ Calif, Ctr Biomol Sci & Engn, Santa Cruz, Ca 95064, USA. Predicting reliable regions in protein sequence alignments. *Bioinformatics* **18:** (2) 306.

Collette A, Six A. 2002. Univ Paris 6, CNRS URA 1961, 25-28 rue Dr Roux, FR-75724 Paris 15, France. ISEApeaks: An Excel platform for GeneScan and Immunoscope data retrieval, management and analysis. *Bioinformatics* **18:** (2) 329.

Conway T, Kraus B, Tucker DL, Smalley DJ, Dorman AF, McKibben L. 2002. Univ Oklahoma, Dept Bot & Microbiol, Norman, Ok 73069, USA. DNA array analysis in a Microsoft® Windows® environment. *Biotechniques* **32:** (1) 110.

Fahnert B, Hahn D, Guthke R. 2002. Hans Knoell Inst Nat Prod Res, Dept Appl Microbiol, Beutenbergstr 11, DE-07745 Jena, Germany. Knowledge-based assessment of gene expression data from chemiluminescence detection. *J Biotechnol* **94:** (1) 23.

Ghosh D, Chinnaiyan AM. 2002. Univ Michigan, Dept Biostat, 1420 Washington Hghts, Ann Arbor, Mi 48109, USA. Mixture modelling of gene expression data from microarray experiments. *Bioinformatics* **18:** (2) 275.

Hatzigeorgiou AG. 2002. Univ Penn, Dept Genet, 418 Guardian Dr, Philadelphia, Pa 19104, USA. Translation initiation start prediction in human cDNAs with high accuracy. *Bioinformatics* **18:** (2) 343.

Hsiao LL, Jensen RV, Yoshida T, Clark KE, Blumenstock JE, Gullans SR*. 2002. *Brigham & Women's Hosp, 65 Landsdowne St, Cambridge, Ma 02139, USA. Correcting for signal saturation errors in the analysis of microarray data. *Biotechniques* **32:** (2) 330.

Husmeier D, Wright F. 2002. Scottish Crop Res Inst, Biomath & Stat Scotland, Dundee DD2 5DA, Scotland. A Bayesian approach to discriminate between alternative DNA sequence segmentations. *Bioinformatics* **18:** (2) 226.

Jonassen I, Eidhammer I, Conklin D, Taylor WR. 2002. Univ Bergen, Dept Informat, NO-5020 Bergen, Norway. Structure motif discovery and mining the PDB. *Bioinformatics* **18:** (2) 362.

Jones L, Moszer I, Cole ST*. 2001. *Inst Pasteur, Serv Informat Sci, Paris, France. Leproma: A *Mycobacterium leprae* genome browser. *Lepr Rev* **72:** (4) 470.

Kanapin A, Apweiler R, Biswas M, Fleischmann W, Karavidopoulou Y, Kersey P, Kriventseva EV, Mittard V, Mulder N, Oinn T, Phan I, Servant F, Zdobnov E. 2002. European Bioinformat Inst, Wellcome Trust Genome Campus, Cambridge CB10 1SD, England. Interactive InterPro-based comparisons of proteins in whole genomes. *Bioinformatics* **18:** (2) 374.

Kozik A, Kochetkova E, Michelmore R. 2002. Univ Calif, Dept Vegetable Crops, Davis, Ca 95616, USA. GenomePixelizer: A visualization program for comparative genomics within and between species. *Bioinformatics* **18:** (2) 335.

McDaniel J, Pillai SD*. 2002. *Texas A&M Univ, Kleberg Ctr, 2472 TAMU, College Station, Tx 77843, USA. Gel alignment and band scoring for DNA fingerprinting using Adobe® Photoshop®. *Biotechniques* **32:** (1) 120.

Messeguer X, Escudero R, Farre D, Nunez O, Martinez J, Alba M*. 2002. *UCL, Dept Immunol & Mol Pathol, 46 Cleveland St, London W1T 4JF, England. PROMO: Detection of known transcription regulatory elements using species-tailored searches. *Bioinformatics* **18:** (2) 333.

Nielsen HB, Knudsen S. 2002. Tech Univ Denmark, Ctr Biol Sequence Anal, Biocentrum, DK-2800 Lyngby, Denmark. Avoiding cross hybridization by choosing nonredundant targets on cDNA arrays. *Bioinformatics* **18:** (2) 321.

Pearl FMG, Lee D, Bray JE, Buchan DWA, Shepherd AJ, Orengo CA. 2002. UCL, Dept Biochem & Mol Biol, Gower St, London WC1E 6BT, England. The CATH extended protein-family database: Providing structural annotations for genome sequences. *Protein Sci* **11:** (2) 233.

Pietrogrande MC, Marchetti N, Dondi F, Righetti PG. 2002. Univ Ferrara, Dept Chem, via L Borsari 46, IT-44100 Ferrara, Italy. Spot overlapping in two-dimensional polyacrylamide gel electrophoresis separations: A statistical study of complex protein maps. *Electrophoresis* **23:** (2) 283.

Schadt EE, Li C, Ellis B, Wong WH. 2001. UCLA, Dept Biomath, Los Angeles, Ca 90024, USA. Feature extraction and normalization algorithms for high-density oligonucleotide gene expression array data. *J Cell Biochem* **84:** (Suppl 37) 120.

Schageman JJ, Basit M, Gallardo TD, Garner HR, Shohet RV. 2002. UT SW Med Ctr, 5323 Harry Hines Blvd, Dallas, Tx 75390, USA. MarC-V: A spreadsheet-based tool for analysis, normalization, and visualization of single cDNA microarray experiments. *Biotechniques* **32:** (2) 338.

Thijs G, Moreau Y, DeSmet F, Mathys J, Lescot M, Rombauts S, Rouze P, De Moor B, Marchal K*. 2002. *KU Leuven, ESAT-SISTA/COSIC, Kasteelpk Arenberg 10, BE-3001 Louvain, Belgium. INCLUSive: INtegrated CLustering, Upstream of Sequence retrieval and motif sampling. *Bioinformatics* **18:** (2) 331.

Toh H, Horimoto K. 2002. Biomol Engn Res Inst, Dept Bioinformat, 6-2-3 Furuedai, Suita, Osaka 565 0874, Japan. Inference of a genetic network by a combined approach of cluster analysis and graphical Gaussian modeling. *Bioinformatics* **18:** (2) 287.

Tsodikov A, Szabo A, Jones D. 2002. Univ Utah, Huntsman Canc Inst, 200 Circle Hope, Salt Lake City, Ut 84112, USA. Adjustments and measures of differential expression for microarray data. *Bioinformatics* **18:** (2) 251.

Wuju L, Momiao X. 2002. Beijing Inst Basic Med Sci, POB 1303, CN-100850 Beijing, Peoples Rep China. Tclass: Tumor classification system based on gene expression profile. *Bioinformatics* **18:** (2) 325.

Yang YH, Dudoit S, Luu P, Lin DM, Peng V, Ngai J, Speed TP*. 2002. *Univ Calif Berkeley, Dept Stat, 367 Evans Hall, Berkeley, Ca 94720, USA. Normalization for cDNA microarray data: A robust composite method addressing single and multiple slide systematic variation (Electronic paper). *Nucleic Acids Res* **30:** (4) E15.

Zhang CT, Wang J, Zhang R. 2002. Tianjin Univ, Dept Phys, CN-300072 Tianjin, Peoples Rep China. Using a Euclid distance discriminant method to find protein coding genes in the yeast genome. *Comput Chem* **26:** (3) 195.

Zhang ZD, Willson RC, Fox GE*. 2002. *Univ Houston, Dept Biol & Biochem, Houston, Tx 77204, USA. Identification of characteristic oligonucleotides in the bacterial 16S ribosomal RNA sequence dataset. *Bioinformatics* **18:** (2) 244.

Copyright © 2002 John Wiley & Sons, Ltd.
